# Proteomics unveils chemical modifications on protein side chains in raw breast meat of broilers (*Gallus gallus*) affected with growth-related myopathies

**DOI:** 10.5713/ab.24.0892

**Published:** 2025-04-28

**Authors:** Yuwares Malila, Sawanya Charoenlappanit, Narumon Phaonakrop, Yanee Srimarut, Sittiruk Roytrakul

**Affiliations:** 1 National Center for Genetic Engineering and Biotechnology (BIOTEC), National Science and Technology Development Agency, Pathum Thani, Thailand

**Keywords:** Broiler (*Gallus gallus*), Chicken Meat, Growth-related Myopathy, Meat Quality, Protein Modification, Proteomics

## Abstract

**Objective:**

This study aimed to investigate how growth-related myopathies influenced chemical modifications formed on amino acid residues of chicken breast proteins.

**Methods:**

Breasts (*pectoralis major*) of commercial broilers (*Gallus gallus*) were classified into “normal”, White Striping (WS)” and “White Striping + Wooden Breast (WS+WB)” groups (n = 9 per group). The meat was subjected to proteomic analysis using a liquid chromatography-tandem mass spectrometry. Differences in abundance of modified sites, including methylated lysine (Lys) and arginine, acetsylated Lys, and oxidized methionine (Met), due to the growth-related myopathies were identified (false discovery rate [FDR]< 0.05). Biological functions of the proteins were analyzed.

**Results:**

Proteomics revealed 185, 105, and 194 modified sites for methylation, Lys acetylation and Met oxidation, respectively. Of 185, 10 sites from seven proteins (TPM1, MYH, MYH1F, DICER1, RCJMB04_5k17, TPI1, and VIM) showed differential abundance in the methylation (FDR<0.05). Seven acetylated Lys sites from five proteins (TPM1, ADHFE1, SPAG9, PCNT, and RCJMB04_5k17) were differentially expressed. The abundance of those sites in normal samples were lower than those of WS samples (FDR<0.05). As for oxidized Met, differential 62 sites were identified (FDR<0.05). The major Met-oxidized protein was MYH. Met oxidation of 40 sites from 22 proteins was increased in WS samples whereas 19 sites of four proteins (MYL11, MYH, MYH1F, and TNNT2) were increased in WS+WB samples. Only four sites from DICER1, LDHA and LDB3 were found in normal samples (FDR<0.05).

**Conclusion:**

The findings shed light on the links between oxidative stress and oxidized Met in the chicken with growth-related myopathies. In addition, methylation and acetylation modifications likely played a role in dynamic cell signaling to maintain cellular activities, particularly metabolism and energy production, against the stress in the affected birds.

## INTRODUCTION

In the past decade, the occurrence of growth-related myopathies, i.e., White Striping (WS) and Wooden Breast (WB), found particularly on chicken breasts, has raised critical concerns in the poultry industry. This is due mainly to an aberrant visual appearance, reduced water holding capacity, and tough texture of the affected meat, particularly ones with the severe lesion [1]. A broad spectrum of meat abnormality was also detected from mildly to moderately affected meat [2], leading to a difficulty to control quality of the meat processed products. Based on an extensive investigation underlying the cause of growth-related myopathies, a growing evidence has pointed out a complex interplay among several biological processes, including metabolism of macromolecules, muscle fiber growth and development, and chronic inflammation [3]. Of those, an oxidative stress has been highlighted as a main trigger of the overall molecular modifications. The deleterious event was hypothesized to be a consequence of a modern poultry breeding in combination of rearing program which focused on maximizing broiler production performance. The massive muscle fiber structure appeared to hinder oxygenation and waste removal, hence an oxidative stress leading to myodegeneration [3]. Indeed, an increase in lipid and protein oxidation was addressed in the WB meat [4,5].

Protein oxidation in the affected breasts was mainly addressed based on the determination of protein carbonyl content [5,6]. In general, protein carbonyl is used as an indicator of protein oxidation [7]. However, the oxidative environment would induce proteins to undergo other chemical modifications such as hydroxylation and nitration of aromatic and aliphatic groups, sulphoxiation of methionine (Met), and nitrosylation of sulfhydryl groups [8]. In muscle foods, an oxidation of amino acid residues depended on source of reactive oxygen species and the presence of prooxidants [8]. The modified proteins could partially unfold and bind to each other, leading to protein aggregation. In contrast, an extreme oxidative attack on the protein backbones could lead to protein fragmentation. Overall, the oxidative modifications may exert adverse impacts on protein functionality [9,10] and digestibility [11]. Based upon their ability to be readily oxidized, Met and cysteine (Cys), the two sulfur-containing amino acids, can serve as important cellular antioxidants [12]. An oxidation of Cys occurs through disulfide bridging between two Cys, forming cystine. Unlike Cys, Met undergoes an oxidation through the addition of an oxygen atom on its sulfur, resulting in Met sulfoxide (MetO), and usually increasing surface hydrophobicity and perturbing conformations of protein molecules [13]. The failure to regulate Met oxidation was associated with development of defects in basement membrane structure of *Drosophila* [14]. In addition to the oxidative modifications, methylation and acetylation of amino acid residues are other post-translational modification of proteins, commonly recognized on the alteration of histone proteins, hence regulating gene expression [15]. Both processes are reversible and later recognized as dynamic cellular signaling for several biological processes [13]. Methylation, an abundance post-translational modification in eukaryotes, is the transfer of methyl groups from S-adenosyl-L-methionine (SAM) to ɛ-amino group of lysine (Lys) and arginine (Arg) catalyzed by Lys methyltransferases, and Arg methyltransferases, respectively [16]. Upon the modification, the methylated proteins acquire a greater hydrophobicity, deviating their binding abilities [17]. In the case of the acetylation, the chemical reaction is evolutionarily conserved and catalyzed by a crosstalk between acetyltransferases and deacetylases. Occurred at Lys, this modification is chemically stable and dependent on the concentrations of two metabolic key compounds, i.e., acetyl-CoA and nicotinamide adenine dinucleotide [17]. Unlike the methylation, acetylation of Lys neutralizes electrostatic charges of at the modified sites. In eukaryotes, post-translational Lys acetylation plays roles as cell signaling in several biological process, including gene regulation and central metabolism [18].

An advance in proteomic technology using a liquid chromatography-tandem mass spectrometry (LC-MS/MS) has enabled a high-throughput identification of chemical modifications on side chains on protein molecules. By employing such technique, previous reports have addressed oxidized Met in porcine myosin [19], methylated Lys and Arg in human cell lines [20], and in cyanobacterium [21]. To our knowledge, however, the changes in chemical modifications on chicken proteins due to growth-related myopathies are not addressed. Therefore, the objective of this study was to investigate the chemical modifications on side chains of chicken breast proteins using proteomic technique. The findings would complement an insight on biological responses against oxidative stress associated with growth-related myopathies.

## MATERIALS AND METHODS

### Samples and sample collection

Breast meat (*pectoralis major*) of commercial broilers (*Gallus gallus*) was collected at the end of a slaughtering line of a local slaughterhouse (Pathum Thani, Thailand). The samples were classified into “normal”, “WS”, and “WS+WB” by a trained staff based on the classification criteria for growth-related myopathies [2]. The abnormal samples used in this experiment must fall into moderate severity level, according to the criteria of Malila et al [2]. The WS category was classified based solely on visual appearance. In brief, the WS meat must show with white lines running parallel to the muscle fibers on the meat surface. The number of the lines was more than 40 or at least one line was thicker than 1.0 mm. The WB category was identified based on a combination of visual monitoring and palpation. The WB meat showed hardened ridges at least 50% of the breast. On the other hand, the normal samples were those showed no visual abnormality or any hardened area. A total of three treatment groups, including “normal”, “WS” and “WS+WB”, with nine biological replicates per group was focused in this study. Each sample was individually packed in a polyethylene bag and transported under 2°C to 4°C from the slaughterhouse to Food Biotechnology Laboratory (BIOTEC, Pathum Thani, Thailand). The meat was excised from the cranial regions, at similar location, of the breast, and stored at −80°C until further analysis.

### Sample preparation for liquid chromatography-mass spectrometry/mass spectrometry

Tissues were ground to powder in liquid nitrogen. The fine powder (100 mg) was mixed with 1 volume of 0.5% sodium dodecylsulfate (SDS), vortexed for 1 h and centrifuged at 10,000×g for 15 min. The supernatant was transferred to a new microtube, mixed thoroughly with 2 volumes of cold acetone, and incubated overnight at -20°C. The mixture was then centrifuged at 10,000×g for 15 min and the supernatant was discarded. The protein pellet was subsequently dried and stored at −80°C until further analysis.

After solubilization in 0.5% SDS, protein concentrations in the collected samples were measured via the Lowry assay, using bovine serum albumin as the calibration standard [22]. For in-solution digestion, five micrograms of each protein sample were dissolved completely in 10 mM ammonium bicarbonate (AMBIC). Disulfide bonds were reduced using 5 mM dithiothreitol in 10 mM AMBIC at 60°C for 1 hour, followed by alkylation of sulfhydryl groups with 15 mM iodoacetamide (IAA) in 10 mM AMBIC for 45 minutes at room temperature in the dark. Protein samples were then digested with sequencing-grade porcine trypsin at a 1:20 enzyme-to-substrate ratio for 16 hours at 37°C. The resulting tryptic peptides were dried using a speed vacuum concentrator and reconstituted in 0.1% formic acid for subsequent nano LC-MS/MS analysis.

### Liquid chromatography-mass spectrometry/mass spectrometry

Tryptic peptide samples were prepared for analysis using the Ultimate 3000 Nano/Capillary LC System (Thermo Scientific, Oxford, UK) coupled to a ZenoTOF 7600 mass spectrometer (SCIEX, Framingham, MA, USA). Briefly, one microliter of peptide digest was pre-concentrated on a μ-Precolumn (300 μm i.d.×5 mm, C18 Pepmap 100, 5 μm, 100 Å; Thermo Scientific, UK) and then separated on a 75 μm I.D.×15 cm analytical column packed with Acclaim PepMap RSLC C18 (2 μm, 100 Å, nanoViper; Thermo Scientific, UK). The column was maintained at 60°C in a thermal controlled oven. Solvent A (0.1% formic acid in water) and solvent B (0.1% formic acid in 80% acetonitrile) were used for elution, with a gradient from 5% to 55% solvent B over 30 minutes at a flow rate of 0.30 μL/min. Throughout all acquisitions, the ZenoTOF 7600 system operated under consistent source and gas conditions, including ion source gas 1 at 8 psi, curtain gas at 35 psi, CAD gas at 7 psi, a source temperature of 200°C, positive polarity, and a spray voltage of 3300 V. In data-dependent acquisition (DDA) mode, the system selected the top 50 precursor ions above an intensity threshold of 150 cps from each MS1 survey for MS/MS analysis, with a dynamic exclusion period of 12 seconds after two MS/MS events. MS2 spectra were collected over an m/z range of 100 to 1800, using a 50 ms accumulation time in the Zeno trap. Collision energy parameters included an 80 V declustering potential, with time bins summed to 8 across all channels and a Zeno trap threshold of 150,000 cps. A 3.0-second cycle time was applied for the top-60 DDA.

### Data analysis

For bioinformatics and quantitative analysis, MaxQuant v2.1.4.0 [23] was utilized with the Andromeda search engine to match MS/MS spectra to the UniProt *Gallus gallus* database (downloaded on 2 March 2024). Standard label-free quantitation parameters were applied, allowing up to two missed cleavages and a mass tolerance of 0.6 Da. Searches were conducted with trypsin specificity, incorporating fixed modifications for Cys carbamidomethylation and variable modifications for Met oxidation and N-terminal acetylation. Peptides were required to contain at least seven amino acids, and proteins were identified based on at least two peptides, including one unique peptide. Protein identification was controlled at a 1% false discovery rate (FDR) using a reversed-sequence decoy approach, with a maximum of five modifications per peptide, referencing the UniProt *Gallus gallus* proteome FASTA file. The ProteinGroups.txt file generated by MaxQuant was subsequently imported into Perseus version 1.6.6.0 [24]. Potential contaminants not associated with any UPS1 proteins were excluded, protein maximum intensities were log2-transformed, and missing values were imputed with a constant value (zero). Data normalization, statistical analysis, and visualization were performed using MetaboAnalyst 6.0 [25]. Modification site detection followed the methodology of [26]. Two-dimensional score plots and cluster heat maps were generated based on partial least squares-discriminant analysis (PLS-DA) in the software. Differences in modified site abundance among different growth-related myopathies were analyzed using one-way analysis of variance (ANOVA) within a completely randomized design, with significant differential modification sites identified by FDR<0.05.

## RESULTS

### Different profiles of methylated lysine and arginine among growth-related myopathies

**Figure 1:**
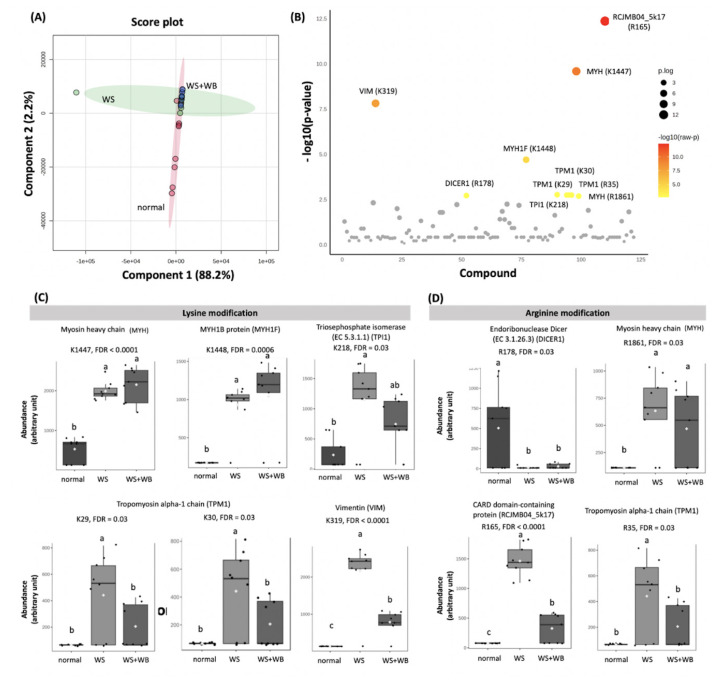
Differential abundance of methylated lysine (K) and arginine (R) residues among proteins of chicken breasts with different growth-related myopathies, i.e., normal, WS, WS+WB. (A) Two-dimension score plots are results of a partial least square-discriminant analysis. (B) Scatter plots indicate modified sites with differential abundance (FDR<0.05) among the samples. Box plots illustrate average abundance (±standard deviation) of (C) methylated lysine and (D) methylated arginine sites among normal, WS, and WS+WB samples. Different letters above bars indicate statistical significance (FDR<0.05). WS, White Striping; WB, Wooden Breast; FDR, false discovery rate.

A PLS-DA score plot (Figure 1A) illustrated that within normal and WS groups, their dataset of were somewhat scattered. An intersection between normal and WS groups suggested that there were methylated sites with comparable abundance among the two treatments. In addition, the WS+WB samples were clustered within such overlapped region. Proteomics reported 185 modified sites of which 10 sites from seven proteins were differentially abundant (FDR<0.05, Figure 1B) among the treatments. Cluster heatmap of those proteins is shown in Supplement 1. Of those proteins (Supplement 2), three were structural components of myofibrillar proteins (i.e., tropomyosin [TPM]1, myosin heavy chain [MYH], and MYH1F), two were regulatory proteins (i.e., DICER1, and RCJMB04_5k17), and the other two proteins were glycolytic enzyme (i.e., TPI1) and intermediate filament (i.e., vimentin [VIM]).

Considering methylated Lys (Figure 1C), the abundance of those modified sites in WS samples were higher than those of normal samples (FDR<0.05). As for WS+WB samples, the abundance of the methylated Lys in MYH (K1447) and MYH1F (K1448) were higher than those of normal samples (FDR<0.05), but did not differ from those of WS samples (FDR≥0.05). In contrast, the methylated Lys abundance on TPM1 (K29, K30) and VIM (K319) in WS+WB samples were lower than those of WS samples (FDR<0.05). Whilst, on TPI1 (K218), its abundance was at intermediate level between normal and WS samples without any statistical significance (FDR≥0.05).

As for Arg methylation (Figure 1D), the modified Arg on DICER1 (R178) in normal samples were higher than that of WS and WS+WB samples (FDR<0.05). At this site, no significant differences between the two groups of abnormal samples were observed (FDR≥0.05). On MYH (R1861) and RCJMB04_5k17 (R165), the meat with growth-related myopathies showed significantly higher abundance than those of normal samples (FDR<0.05). As for the site on TPM1 (R35), an increased methylated Arg was observed in WS samples compared to the others (FDR<0.05).

### Different profiles of acetylated lysine among growth-related myopathies

**Figure 2:**
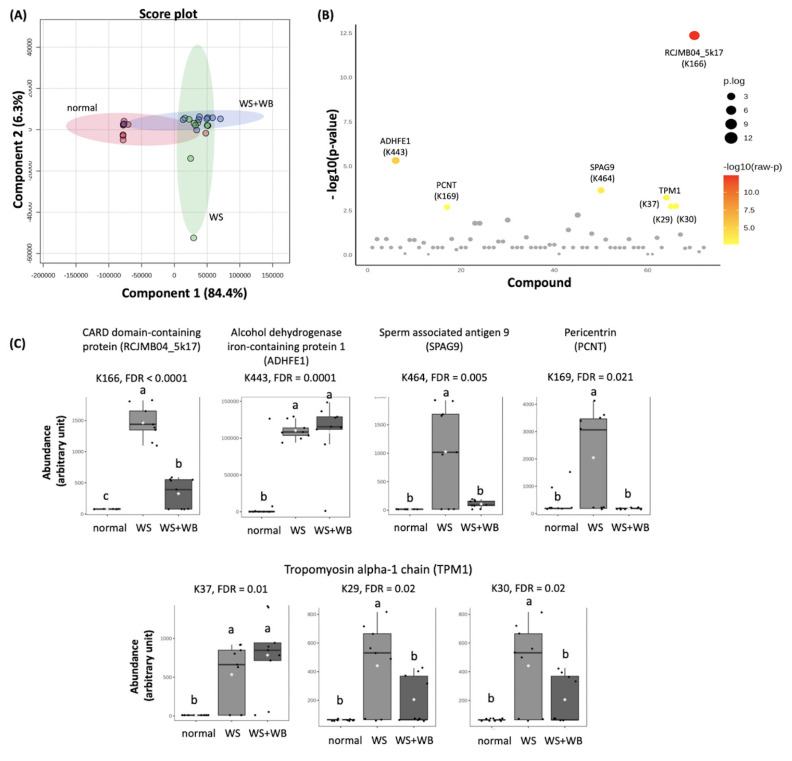
Differential abundance of aceylated lysine (K) residues among proteins of chicken breasts with different growth-related myopathies, i.e., normal, WS, WS+WB. (A) Two-dimension score plots are results of a partial least square-discriminant analysis. (B) Scatter plots indicate modified sites with differential abundance (FDR<0.05) among the samples. (C) Box plots illustrate average abundance (±standard deviation) of acetylated lysine (K) sites among normal, WS, and WS+WB samples. Different letters above bars indicate statistical significance (FDR<0.05). WS, White Striping; WB, Wooden Breast; FDR, false discovery rate.

A PLS-DA score plot of acetylated Lys is shown in Figure 2A. The plot indicated a close clustering between normal and each abnormal group. In addition, it was difficult to distinguish between WS and WS+WB groups. Among 105 acetylated Lys sites identified (Supplement 3), seven sites from five proteins (Figure 2B; Supplement 4) were differentially abundant (FDR<0.05). Those five proteins included one structural protein (i.e., TPM1), three catalytic enzymes (i.e., ADHFE1, SPAG9, pericentrin (PCNT)), and one regulatory protein (i.e., RCJMB04_5k17). As shown in Figure 2C, the abundance of those acetylated Lys sites in normal samples were lower than those of WS samples (FDR<0.05). No significant differences (FDR≥0.05) between WS and WS+WB samples were observed for ADHFE1 (K443) and TPM1 (K37). On the other hand, the acetylated Lys on RCJMB0$_5k17 (K165), SPAG9 (K464), PCNT (K169) and TPM1 (K29, and K30) were detected at a lesser extent in WS+WB compared with those of WS samples (FCR<0.05).

### Different profiles of oxidized methionine among growth-related myopathies

**Figure 3:**
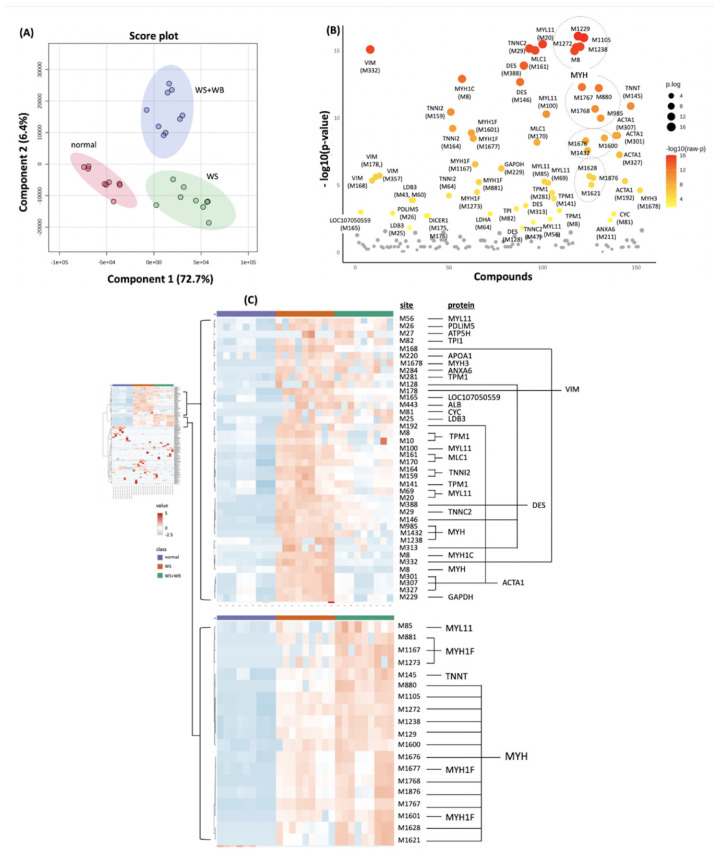
Differential abundance of oxidize methionine (M) residue among proteins of chicken breasts with different growth-related myopathies, i.e., normal, WS, WS+WB. (A) Two-dimension score plots are results of a partial least square-discriminant analysis. (B) Scatter plots indicate modified sites with differential abundance (false discovery rate<0.05) among the samples. (C) Cluster heat maps illustrate sites of oxidized methionine and their associated proteins.

Based on a PLS-DA score plot (Figure 3A), the disparity of oxidized Met profiles among the normal, WS and WS+WB samples was apparent. Proteomic analysis revealed 194 compounds with oxidative Met modification. Of those, 62 sites were differentially expressed among the treatments (Figure 1B; Supplement 2), a majority of proteins with oxidative Met modification sites are muscle structural proteins of thick and thin filaments (e.g., MYH, alpha actin, troponin C; TNNC, troponin T; TNNT, TPM), followed by intermediate filaments (i.e., VIM, and desmin; DES). In addition to those, the Met side chains on the key glycolytic enzymes, (i.e., GAPDH, TPI1, LOC107050559, and LDHA, endoribonuclease (i.e., DICER1), cytochrome C (CYC), annexin A6 (ANXA6), and regulatory proteins (i.e., PDLIM5, LDB3) were also undergone oxidation (Supplement 3). As shown by a cluster heat map (Figure 3C), Met oxidation of 40 sites from 22 proteins was increased in WS samples whereas 19 sites of four proteins (i.e., MYL11, MYH, MYH1F, and TNNT2) were increased in WS+WB samples. Only four sites from DICER1 (M175, M176), LDHA (M306), LDB3 (M43, M60) of which increased Met oxidation was found in normal samples (FDR<0.05, Supplement 5).

**Figure 4:**
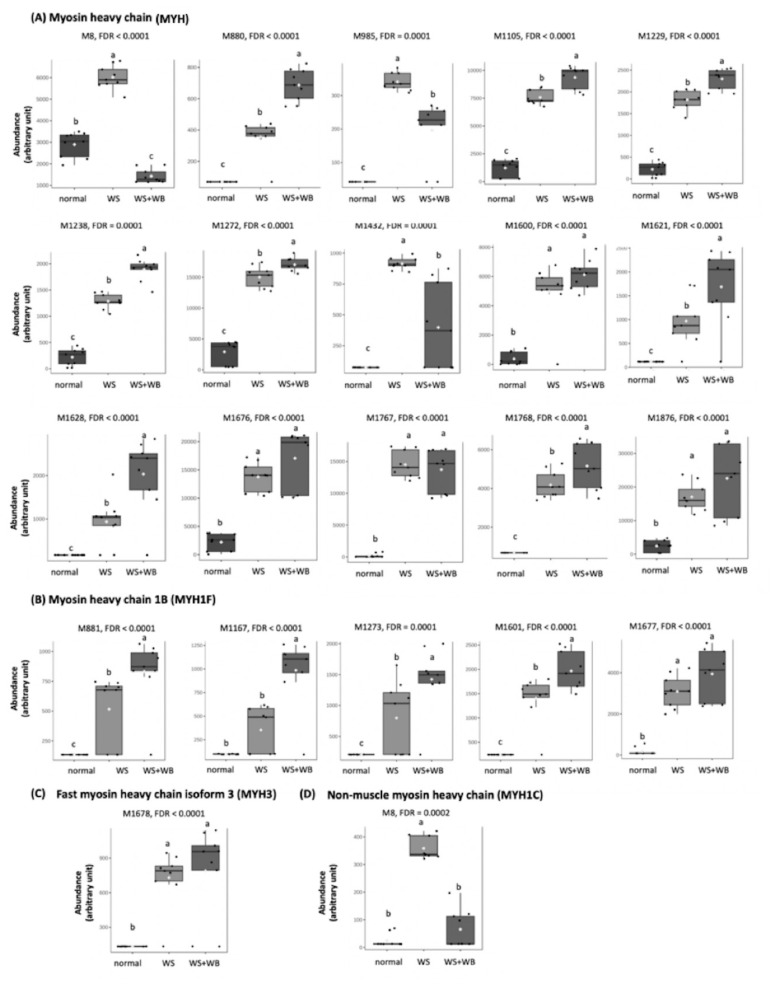
Box plots illustrate average abundance (±standard deviation) of oxidative methionine (M) sites in proteins associated with myosin heavy chains of chicken meat exhibiting normal, wooden breast (WB), white striping (WS), and WS+WB. Different letters above bars indicate statistical significance (FDR<0.05). FDR, false discovery rate.

The majority of the differential oxidized Met profiles were observed on MYH (Supplement 6; Figure 4A) with more sites located towards the end of the polypeptide chain (Supplement 7). The abundance of oxidized Met on most differentially abundant sites of most sites, except for the M8 site, were lower in normal compared to WS and WS+WB samples (FDR< 0.05). An additional isoforms of MYH were identified (Figures 4B–4D). As for MYH1F (Figure 4B), the abundance of oxidized Met at M881, M1273, M1601 and M1677 were higher in the abnormal samples compared with those of normal groups (FDR<0.05). On those modified sites, the abundance of WS samples were lower than that of WS+WB samples (FDR<0.05), except for M1677 of which expression in WS and WS+WB did not differ (FDR≥0.05). At M1167 of MYH1F, the abundance of oxidized Met was not different between normal and WS samples (FDR≥0.05) but the levels among those two samples were lower than that of WS+WB samples (FDR<0.05). The differential Met oxidized pattern on M1678 of MYH3 (Figure 4C) was similar to that on M1677 of MYH1F. In contrast, on M8 of MYH1C, oxidized Met abundance of WS samples were higher than that of normal and WS+WB samples (FDR<0.05) whereas the last two showed no difference among each other (FDR≥0.05).

**Figure 5:**
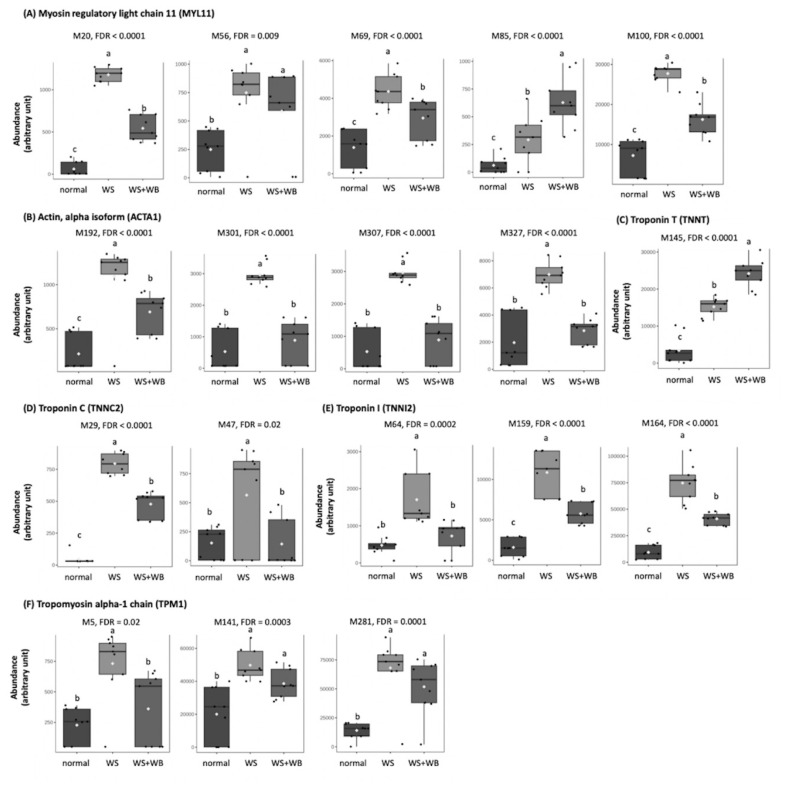
Box plots illustrate average abundance (±standard deviation) of oxidative methionine (M) sites in proteins associated with muscle fiber thick filaments (i.e., (A) myosin regulatory light chain, (B) actin, (C) troponin T, (D) troponin C, (E) troponin C, (E) troponin I, and (F) tropomyosin) of chicken meat exhibiting normal, wooden breast (WB), white striping (WS), and WS+WB. Different letters above bars indicate statistical significance (FDR<0.05). FDR, false discovery rate.

Considering the other protein components of thick filaments (Figure 5), similar to those found in MYH, abundance of oxidized Met found on MYL11 (Figure 5A) in normal samples were lower than those of abnormal samples (FDR< 0.05). As for alpha-actin (Figure 5B), the oxidized Met in WS samples showed the highest abundance compared with normal and WS+WB samples (FDR<0.05). However, no different abundances of modified M301, M307 and M327 were observed between normal and WS+WB samples (FDR≥0.05). As shown in Figure 5C, differential abundance of TNNT was identified only on M145 in which WS+WB samples showed the greatest extent, followed by WS, and normal samples, respectively (FDR<0.05). Two differential oxidative sites were observed on TNNC (Figure 5D). On M29, WS sample exhibited the highest abundance of the oxidized site, followed by WS+WB, and normal samples, respectively (FDR<0.05). On the other hand, the abundance of modified M47 in WS was higher than those of normal and WS+WB samples (FDR< 0.05) but the latter two showed no difference between each other (FDR≥0.05). The differential patterns found on TNNI2 (Figure 5E), and the modified M5 of TPM1 (Figure 5F) were similar to those found on TNNT in which the abundances found in WS samples were at the greater extent compared with normal and WS+WB samples. As for M141 and M281 of TPM1, the abundance of modified sites in the abnormal samples were higher than those of normal ones (FDR<0.05).

**Figure 6:**
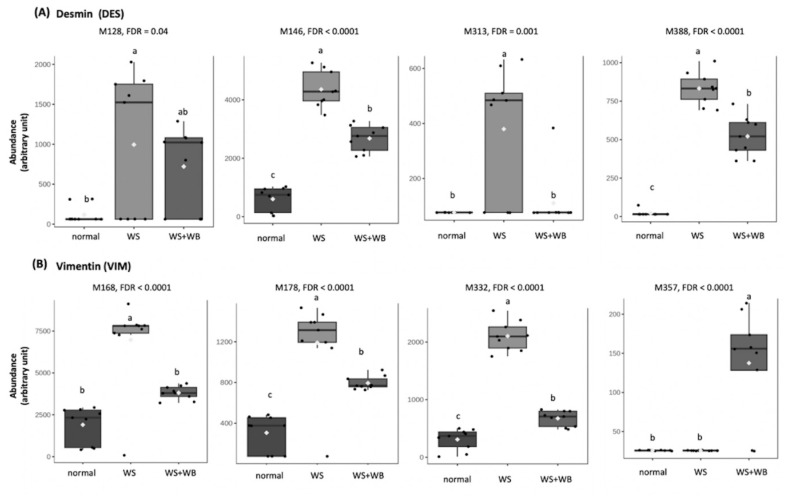
Box plots illustrate average abundance (±standard deviation) of oxidative methionine (M) sites in extracellular-matrix proteins, including (A) desmin and (B) vimentin, of chicken meat exhibiting normal, white striping (WS), and white striping+wooden breast (WS+WB). Different letters above bars indicate statistical significance (FDR<0.05). FDR = false discovery rate.

Interestingly, differential abundance of oxidized sites on two intermediate filaments, including DES (Figure 6A) and VIM (Figure 6B), were identified. Focusing on DES, WS samples showed the higher abundance at oxidized M146, M313, and M388 than the other samples (FDR<0.05). In contrast, the abundance of oxidized M128 was not different between WS and WS+WB samples (FDR 0.05) but the expression of those two were higher than that of normal samples (FDR< 0.05).

## DISCUSSION

In this study, the chemical modifications of amino acid residues on polypeptide chains were analyzed and compared among the meat with different growth-related myopathies. Based on proteomic analysis, Lys and Arg methylation, Lys acetylation, and Met oxidation were in the current focus according to their biological roles in stress responses [12,16,18]. Overall, this proteomic-based study revealed distinct patterns of protein modification in chicken meat associated with different growth-related myopathies. However, it is worth noting that whether modifications were the causes or responses of the oxidative stress requires further mechanistic investigations.

In general, Lys and Arg methylation and Lys acetylation are common post-translational modifications. First identified in histone modifications for regulating gene expression, such modifications have later observed as tailor protein activities controlling metabolic abundances [17]. This might explain the differences in abundances of methylated events on endoribonuclease DICER1, The CARD domain (i.e., RCJMB04_ 5k17), and TPI1. In addition, the CARD domain facilitates protein-protein interactions when the cells are stress, hence activating downstream apoptotic and informatory response [27]. The current results align well with the defective energy production along with a tissue ATPase deficiency in the birds with growth-related myopathies [28]. As for the structural components of the muscle fibers, methylated lysine on MYH has been identified since 1960s [29]. So far, the *in vivo* functional roles of such modification were inadequately discussed. Still, the report of Phan et al [30] unveiled that the methylated lysine of MYH accelerated nucleotide dissociation from myosin, altered the coupling between myosin and actin, and impaired in vitro actin mobility over the myosin. In this regard, the methylation might involve in energy preservation from ATP depletion in the affected meat. In the methylation, SAM is a major methyl donor, leading to a formation of S-adenosylhomocysteine (SAH). The decreased ratio SAM and SAH ratio was addressed in the patients with hemodialysis failure. In fact, a 11-fold increased SAH was observed in the broilers with severe WB condition. The current findings of increased methylated Lys and Arg in the affected birds might partly explain such knowledge gaps in the growth-related myopathies. In addition, the lower abundance of methylated Lys on TPM1 (K29 and K30) and on VIM (K319) observed in WS+WB samples compared with WS samples suggested the links between such modifications and myopathic severity. Another possible explanation is that such decreases in the modifications might be related with stress adaptation among the birds affected with WS+WB condition.

The current proteomic-based analysis indicated that the proteins exhibiting differential acetylated Lys were mainly located in the cytoplasmic compartment. Most of the proteins appeared to associate with stress-response mechanisms. For example, ADHFE1 is responsible for catalyzing a reversible oxidation of 4-hydroxybutyrate, a lipid-derived ketone body produced by liver under carbohydrate starvation, supporting glucolipitoxicity in the myopathic birds. The Jun-N-terminal kinase-interacting protein (i.e., SPAG9) play roles in trafficking lysosomal positioning during stress-activated kinase systems. PCNT, an integral component of the centrosomes, mediating microtubule orientation during the cell cycles. In the case of TPM1, previous study showed that the acetylation at TPM1 N-terminus contributed to conformational changes, mediating kinetic properties of actin-tropomyosin-myosin complexes. In this study, the acetylation was observed on K29, K30 and K37, which might also plausibly affect the interactions between the thick and thin filaments during development of the growth-related myopathies. However, this aspect required further investigation.

Among the three modification events, differential occurrences of Met oxidation were the most identified among the myopathies, showing MYH as the major modified protein. Out of the 14 identified sites on MYH (Figure 4A), 13 oxidized Met sites, except for M8, exhibited significantly higher abundant in the abnormal samples than that of the normal group (FDR<0.05). The abundance tended to increase when WS was accompanied with WB lesions. The findings strongly supported an intracellular oxidative stress induced within the gigantic muscle fiber of the modern broilers [3]. The current results were well aligned with previous studies identifying Met oxidation in stress-induced human Jurkat cells and mice with an experimentally-induced severe septic shock. Recently, Yin et al [26] addressed malondialdehyde-induced chemical modifications, including Schiff-based and dihydropyridine (DHP)-type adducts, on beef myofibrillar protein. In the previous study, myosin-1 was also the major modified proteins with more modifications localized towards the tail region. Despite the different modified residues, the current investigation was well corresponded with such report of Yin et al [26]. Hence, our findings supported the susceptibility of the MYH tail regions to an oxidative condition. This was due potentially to the enriched polar residues of this region [10]. In addition to myosin-1, Yin et al, [26] also observed chemical modifications on other myofibrillar proteins, including TPM1, TPM-beta chain, TNNT, TNNI2, MYL and actin. Except for the TPM-beta chain, similar proteins on thick and thin filaments were identified with differential oxidized Met profiles in the present study. Oxidation of the sulfur-containing amino acids can be reversed by MetO reductase system. Therefore, it was reasonable to hypothesize that Met oxidation in the abnormal samples might play part into cellular protective mechanisms against oxidative stress [10]. However, it is worth noting that the current study could not differentiate between oxidative damage and regulatory Met oxidation. Further investigation is indeed required to elucidate on this aspect.

Unlike on the MYH, the oxidized Met abundances on actin, TNNC,2 TNNI2, and TPM1 (Figure 5) were the most pronounced in WS samples. An explanation on such deviated patterns remained unclear. The results, however, led us to two further hypotheses. First, the current observation appeared to support the different etiologies between WS and WS+WB phenotypes. Secondly, a profound Met oxidation on WS+WB samples might induce a redistribution of MYH structural states, including a strong actin-binding transition, shifting the availability of Met residues on the thin filaments.

In addition to the protein components of thick and thin filament, proteomics identified differential patterns of modified DES and VIM. The two proteins are the members of intermediate filaments, providing the supporting roles in myofibril alignment and organization, essentially during muscle regeneration. Increases in DES and VIM at both transcriptional and protein levels were addressed in WS and WB chicken breast muscles, respectively, suggesting the intense regenerative process within skeletal muscle of the affected birds. Herein, the occurrence of oxidized Met on DES and VIM as well as methylated Lys on VIM were more pronounced in the abnormal samples. The greater rise of the modified proteins was associated with the occurrence of WS condition. In this regard, it could be speculated that the modified residues might induce structural changes and impair their muscle regenerative functions [13], hence disrupting muscle cytoarchitecture, in the affected birds.

Another aspect is worth highlighting. Due to the lack of previous studies in poultry, the discussion regarding Met oxidation was based mainly upon the studies of human and murine models. The physiological divergence among the species should be considered. While broilers prioritized growth-driven metabolisms, mammals emphasize homeostasis and epigenetic regulation. However, conserved biochemical mechanisms of Met oxidation and impacts on protein functionalities were observed across chickens, human and mice [12–15].

A decrease in digestibility of fish myofibrillar proteins was shown to tightly related with malondialdehyde-induced modifications at Lys and Arg resided nearby the proteolytic cleavage sites [11]. In addition, the modified proteins were retained in the in vitro everted-rat-gut-sac, suggesting a reduced protein absorbability [11]. Our previous studies demonstrated a greater degree of in vitro protein digestibility of cooked chicken meat with growth-related myopathies than that of the normal samples. An extensive muscle fiber fragmentation in the abnormal samples due to myodegeneration appeared to assist the in vitro proteolytic digestion [3]. However, the changes in protein absorbability due to the myopathies have not yet investigated. Whether the modifications would be carried over to cooked state of the abnormal meat and exerted any impacts on protein absorbability remained to be elucidated.

In conclusion, the current proteomic study pinpointed increased counts of chemical modifications (i.e., methylated Lys and Arg, acetylated Lys and oxidized Met) on polypeptide chains of chicken breast meat with growth-related myopathies. The major differential modification profile was Met oxidation, supporting an extensive cellular oxidative stress associated with the myopathies. The abundant differences in methylation of Lys and Arg along with Lys acetylation were mainly observed among enzymes and regulatory proteins, suggesting the roles of those modifications as part of cellular mechanisms associated with growth-related myopathies. Protein components of thick and think filaments, particularly the tail region of MYH, were the most affected with Met oxidation. Whist, all modifications were observed for TPM1. A comprehensive insight obtained from this study would lay a foundation for mitigation strategies from production management to nutritional intervention to minimize the oxidative stress triggered myopathies. Further in-depth investigation is warranted to assure the nutritional quality of the chicken meat.

## SUPPLEMENTARY MATERIAL

**Supplement 1**. Cluster heat maps of relative abundance of methylated lysine (K) and arginine (R) according to growth-related myopathies in chicken breasts.

**Supplement 2**. Differential methylation sties identified among the protein of chicken breast meat exhibiting growth-related myopathies

**Supplement 3**. Cluster heat maps of relative abundance of acetylated lysine (K) according to growth-related myopathies in chicken breasts.

**Supplement 4**. Differential acetylated lysine sites identified among the protein of chicken breast meat exhibiting growth-related myopathies.

**Supplement 5**. Box plots illustrate average abundance (±standard deviation) of oxidative methionine (M) sites in proteins isolated from chicken meat exhibiting normal, wooden breast (WB), white striping (WS), and WS+WB.

**Supplement 6**. Number of oxidative methionine sties differentially identified among the protein of chicken breast meat exhibiting growth-related myopathies.

**Supplement 7**. Amino acid sequence of myosin heavy chains, skeletal muscle (Unipro_P13538). Yellow highlight indicates oxidized methionine sites with differential abundance (false discovery rate, FDR<0.05) among the chicken breast with different growth-related myopathies (i.e., normal, White Stirping (WS), WS+Wooden Breast (WB)).
