# Supplementary material for: Proteomics unveils chemical modifications on protein side chains in raw breast meat of broilers (Gallus gallus) affected with growth-related myopathies
Source: Anim Biosci. 2025 Apr 28;38(9):2008–20. doi: 10.5713/ab.24.0892 (PMC12415449; doi:10.5713/ab.24.0892)
Supplement: Supplementary file 2 [file ab-24-0892-Supplementary-2.pdf]

Supplement 2. Differential methylation sties identified among the protein of chicken breast meat exhibiting growth-related myopathies

Protein ID	Relevant KEGG biological processes	Number of sites	Modification sites
**Thick and think filaments (6)**			
TPM1	actin filament organization [GO:0007015]; cardiac muscle contraction [GO:0060048]	3	K29, K30, R35
MYH	muscle contraction [GO:0006936]	2	K1447, R1861
MYH1F	muscle contraction [GO:0006936]	1	K1448
**Regulatory proteins (2)**			
DICER1	cellular component organization [GO:0016043]; pre-miRNA processing [GO:0031054]; siRNA processing [GO:0030422]	1	R178
RCJMB04_5k17	activation of NF-kappaB-inducing kinase activity [GO:0007250]; B cell receptor signaling pathway [GO:0050853]; positive regulation of canonical NF-kappaB signal transduction [GO:0043123]; regulation of apoptotic process [GO:0042981]	1	R165
**Glycolytic enzyme (1)**			
TPI1	canonical glycolysis [GO:0061621]; gluconeogenesis [GO:0006094]; glyceraldehyde-3-phosphate biosynthetic process [GO:0046166]; glycerol catabolic process [GO:0019563]; glycolytic process [GO:0006096]; methylglyoxal biosynthetic process [GO:0019242]	1	K218
**Intermediate filament (1)**			
VIM	intermediate filament organization [GO:0045109]; intermediate filament polymerization [GO:0045107]; skeletal muscle organ development [GO:0060538]	1	K319
	**Total**	10	
