# Supplementary material for: Proteomics unveils chemical modifications on protein side chains in raw breast meat of broilers (Gallus gallus) affected with growth-related myopathies
Source: Anim Biosci. 2025 Apr 28;38(9):2008–20. doi: 10.5713/ab.24.0892 (PMC12415449; doi:10.5713/ab.24.0892)
Supplement: Supplementary file 3 [file ab-24-0892-Supplementary-3.pdf]

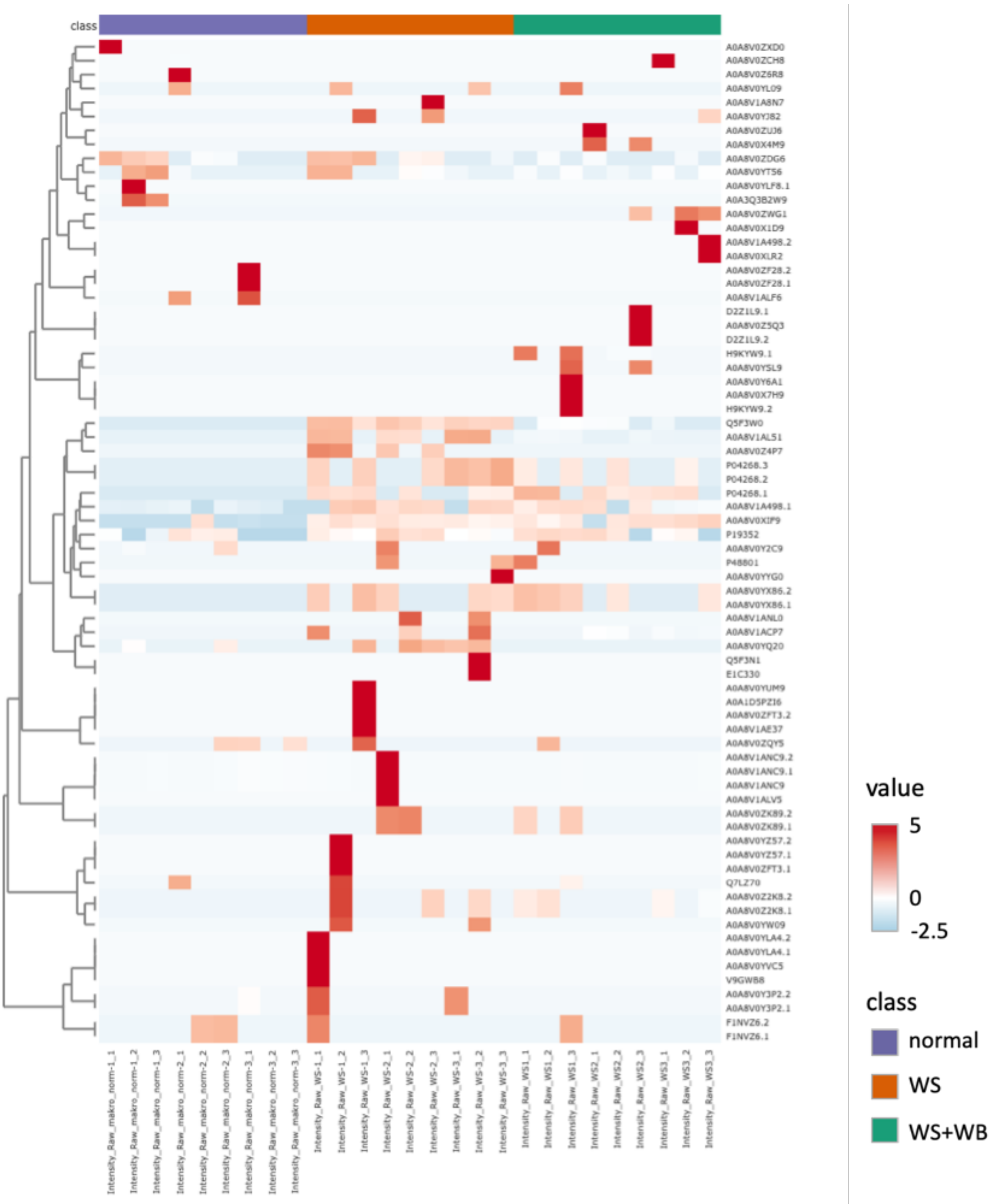

Supplement 3. Cluster heat maps of relative abundance of acetylated lysine (K) according to growth-related myopathies in chicken breasts. Growth-related myopathies include normal, White Striping; WS, White Striping and Wooden Breast (WB). Oxidative modification was focused on methionine side chain of chicken meat proteins.
