# Supplementary material for: Proteomics unveils chemical modifications on protein side chains in raw breast meat of broilers (Gallus gallus) affected with growth-related myopathies
Source: Anim Biosci. 2025 Apr 28;38(9):2008–20. doi: 10.5713/ab.24.0892 (PMC12415449; doi:10.5713/ab.24.0892)
Supplement: Supplementary file 4 [file ab-24-0892-Supplementary-4.pdf]

Supplement 4. Differential acetylated lysine sites identified among the protein of chicken breast meat exhibiting growth-related myopathies

protein ID	Gene Ontology (biological process)	Number of sites	Modification sites
**Thick and think filaments (3)**			
TPM1	actin filament organization [GO:0007015]; cardiac muscle contraction [GO:0060048]	3	K29, K30, K37
**Catalytic enzymes (2)**			
ADHFE1	Hydroxyacid-oxoacid transhydrogenase activity; lipid metabolic process; Responsible for the oxidation of 4-hydroxybutyrate in mammalian tissues	1	K443
SPAG9	MAP-kinase scaffold activity [GO:0005078]; lysosome localization; negative regulation of protein phosphorylation; positive regulation of cell migration; positive regulation of MAPK cascade	1	K464
**Cell cycle / cell division (1)**			
PCNT	signal transduction [GO:0007165]	1	K169
**Regulatory proteins (1)**			
RCJMB04_5 k17	activation of NF-kappaB-inducing kinase activity [GO:0007250]; B cell receptor signaling pathway [GO:0050853]; positive regulation of canonical NF-kappaB signal transduction [GO:0043123]; regulation of apoptotic process [GO:0042981]	1	K166
	**Total**	**7**	
