# Supplementary material for: Proteomics unveils chemical modifications on protein side chains in raw breast meat of broilers (Gallus gallus) affected with growth-related myopathies
Source: Anim Biosci. 2025 Apr 28;38(9):2008–20. doi: 10.5713/ab.24.0892 (PMC12415449; doi:10.5713/ab.24.0892)
Supplement: Supplementary file 6 [file ab-24-0892-Supplementary-6.pdf]

Supplement 6. Number of oxidative methionine sties differentially identified among the protein of chicken breast meat exhibiting growth-related myopathies

Protein ID	Relevant KEGG biological processes	Number of sites
**Key structural components of thick and thin filaments (40)**		
MYH	muscle contraction [GO:0006936]	15
MYH1F	muscle contraction [GO:0006936]	5
MYL11	muscle contraction [GO:0006936]; skeletal muscle tissue development [GO:0007519]	5
ACTA1	skeletal muscle fiber development [GO:0048741]; skeletal muscle thin filament assembly [GO:0030240]	4
LDB3	Muscle fiber integrity	3
TPM1	actin filament organization [GO:0007015]	3
TNNI2	regulation of muscle contraction [GO:0006937]	3
MLC1	regulation of myosin II filament assembly [GO:0043520]	2
TNNC2	skeletal muscle contraction [GO:0003009]	2
MYH3	muscle contraction [GO:0006936]	1
MYH1C	muscle contraction [GO:0006936]	1
TNNT	regulation of muscle contraction [GO:0006937]	1
**Intermediate filaments (8)**		
VIM	intermediate filament organization [GO:0045109]; intermediate filament polymerization [GO:0045107]; skeletal muscle organ development [GO:0060538]	4
DES	intermediate filament organization [GO:0045109]; intermediate filament polymerization [GO:0045107]; skeletal muscle organ development [GO:0060538]	4
**Glycolytic enzymes (4)**		
GAPDH	gluconeogenesis [GO:0006094]; glycolytic process [GO:0006096]; microtubule cytoskeleton organization [GO:0000226]; negative regulation of apoptotic process [GO:0043066]	1
TPI1	canonical glycolysis [GO:0061621]; gluconeogenesis [GO:0006094]; glyceraldehyde-3-phosphate biosynthetic process [GO:0046166]; glycerol catabolic process [GO:0019563]; glycolytic process [GO:0006096]; methylglyoxal biosynthetic process [GO:0019242]	1
LOC10705055[illegible]	glycolytic process [GO:0006096]	1
LDHA	lactate metabolic process [GO:0006089]; pyruvate metabolic process [GO:0006090]	1
**Post-transcriptional modification (2)**		
DICER1	cellular component organization [GO:0016043]; pre-miRNA processing [GO:0031054]; siRNA processing [GO:0030422]	2
**Family of calcium-dependent membrane and phospholipid binding proteins (1)**		
ANXA6	apoptotic signaling pathway [GO:0097190]; calcium ion homeostasis [GO:0055074]; mitochondrial calcium ion homeostasis [GO:0051560]; negative regulation of sequestering of calcium ion [GO:0051283]; plasma membrane repair [GO:0001778]; regulation of muscle contraction [GO:0006937]	1
**Oxidative respiration (1)**		
CYC	mitochondrial electron transport, cytochrome c to oxygen [GO:0006123]; mitochondrial electron transport, ubiquinol to cytochrome c [GO:0006122]	1
**Regulatory protein (1)**		
PDLIM5	skeletal muscle fiber development [GO:0048741]	1
	**Total**	**62**
