# Supplementary material for: Proteomics unveils chemical modifications on protein side chains in raw breast meat of broilers (Gallus gallus) affected with growth-related myopathies
Source: Anim Biosci. 2025 Apr 28;38(9):2008–20. doi: 10.5713/ab.24.0892 (PMC12415449; doi:10.5713/ab.24.0892)
Supplement: Supplementary file 7 [file ab-24-0892-Supplementary-7.pdf]

Length 1,939

Mass (Da) 223,145

Last updated 2007-01-23 v4

Checksum[i] F6DAD73CABD82BFD

```
        10         20         30         40         50         60         70         80         90
MASPDAEMAA FGEAAPYLRK SEKERIEAQN KPFDAKSSVF VVHPKESFVK GTIQSKEGGK VTVKTEGGET LTVKEDQVFS MNPPKYDKIE

       100        110        120        130        140        150        160        170        180
DMAMMTHLHE PAVLYNLKER YAAWMIYTYS GLFCVTVNPY KWLPVYNPEV VLAYRGKKRQ EAPPHIFSIS DNAYQFMLTD RENQSILITG

       190        200        210        220        230        240        250        260        270
ESGAGKTVNT KRVIQYFATI AASGEKKKEE QSGKMQGTLE DQIISANPLL EAFGNAKTVR NDNSSRFGKF IRIHFGATGK LASADIETYL

       280        290        300        310        320        330        340        350        360
LEKSRVTFQL PAERSYHIFY QIMSNKKPEL IDMLLITTNP YDYHYVSQGE ITVPSIDDQE ELMATDSAID ILGFSADEKT AIYKLTGAVM

       370        380        390        400        410        420        430        440        450
HYGNLKFKQK QREEQAEPDG TEVADKAAYL MGLNSAELLK ALCYPRVKVG NEFVTKGQTV SQVHNSVGAL AKAVYEKMFL WMVIRINQQL

       460        470        480        490        500        510        520        530        540
DTKQPRQYFI GVLDIAGFEI FDFNSFEQLC INFTNEKLQQ FFNHHMFVLE QEEYKKEGIE WEFIDFGMDL AACIELIEKP MGIFSILEEE

       550        560        570        580        590        600        610        620        630
CMFPKATDTS FKNKLYDQHL GKSNNFQKPK PAKGKAEAHF SLVHYAGTVD YNISGWLEKN KDPLNETVIG LYQKSSVKTL ALLFATYGGE

       640        650        660        670        680        690        700        710        720
AEGGGGKKGG KKKGSSFQTV SALFRENLNK LMANLRSTHP HFVRCIIPNE TKTPGAMEHE LVLHQLRCNG VLEGIRICRK GFPSRVLYAD

       730        740        750        760        770        780        790        800        810
FKQRYRVLNA SAIPEGQFMD SKKASEKLLG SIDVDHTQYR FGHTKVFFKA GLLGLLEEMR DDKLAEIITR TQARCRGFLM RVEYRRMVER

       820        830        840        850        860        870        880        890        900
RESIFCIQYN VRSFMNVKHW PWMKLFFKIK PLLKSAESEK EMANMKEEFE KTKEELAKSE AKRKELEEKM VVLLQEKNDL QLQVQAEADS

       910        920        930        940        950        960        970        980        990
LADAEERCDQ LIKTKIQLEA KIKEVTERAE DEEEINAELT AKKRKLEDEC SELKKDIDDL ELTLAKVEKE KHATENKVKN LTEEMAVLDE

      1000       1010       1020       1030       1040       1050       1060       1070       1080
TIAKLTKEKK ALQEAHQQTL DDLQVEEDKV NTLTKAKTKL EQQVDDLEGS LEQEKKLRMD LERAKRKLEG DLKLAHDSIM DLENDKQQLD

      1090       1100       1110       1120       1130       1140       1150       1160       1170
EKLKKKDFEI SQIQSKIEDE QALGMQLQKK IKELQARIEE LEEEIEAERT SRAKAEKHRA DLSRELEEIS ERLEEAGGAT AAQIEMNKKR

      1180       1190       1200       1210       1220       1230       1240       1250       1260
EAEFQKMRRD LEEATLQHEA TAAALRKKHA DSTAELGEQI DNLQRVKQKL EKEKSELKME IDDLASNMES VSKAKANLEK MCRTLEDQLS

      1270       1280       1290       1300       1310       1320       1330       1340       1350
EIKTKEEQNQ RMINDLNTQR ARLQTETGEY SRQAEEKDAL ISQLSRGKQG FTQQIEELKR HLEEEIKAKN ALAHALQSAR HDCELLREQY

      1360       1370       1380       1390       1400       1410       1420       1430       1440
EEEQEAKGEL QRALSKANSE VAQWRTKYET DAIQRTEELE EAKKKLAQRL QDAEEHVEAV NAKCASLEKT KQRLQNEVED LMVDVERSNA

      1450       1460       1470       1480       1490       1500       1510       1520       1530
ACAALDKKQK NFDKILAEWK QKYEETQTEL EASQKESRSL STELFKMKNA YEESLDHLET LKRENKNLQQ EIADLTEQIA EGGKAVHELE

      1540       1550       1560       1570       1580       1590       1600       1610       1620
KVKKHVEQEK SELQASLEEA EASLEHEEGK ILRLQLELNQ IKSEIDRKIA EKDEEIDQLK RNHLRIVESM QSTLDAEIRS RNEALRLKKK

      1630       1640       1650       1660       1670       1680       1690       1700       1710
MEGDLNEMEI QLSHANRMAA EAQKNLRNTQ GTLKDTQIHL DDALRTQEDL KEQVAMVERR ANLLQAEVEE LRGALEQTER SRKVAEQELL

      1720       1730       1740       1750       1760       1770       1780       1790       1800
DATERVQLLH TQNTSLINTK KKLETDIVQI QSEMEDTIQE ARNAEEKAKK AITDAAMMAE ELKKEQDTSA HLERMKKNMD QTVKDLHVRL

      1810       1820       1830       1840       1850       1860       1870       1880       1890
DEAEQLALKG GKKQLQKLEA RVRELEGEVD SEQKRSAEAV KGVRKYERRV KELTYQCEED RKNILRLQDL VDKLQMKVKS YKRQAEEAEE

      1900       1910       1920       1930
LSNVNLSKFR KIQHELEEAE ERADIAESQV NKLRVKSREI HGKKIEEEE
```

Supplement 7. Amino acid sequence of myosin heavy chain, skeletal muscle (Unipro_P13538). Yellow highlight indicates oxidized methionine sites with differential abundance (false discovery rate, FDR < 0.05) among the chicken breast with different growth-related myopathies (i.e., normal, White Stirping (WS), WS+Wooden Breast (WB)). The red line separates myosin head and tail regions.
