# Supplementary material for: Proteomics unveils chemical modifications on protein side chains in raw breast meat of broilers (Gallus gallus) affected with growth-related myopathies
Source: Anim Biosci. 2025 Apr 28;38(9):2008–20. doi: 10.5713/ab.24.0892 (PMC12415449; doi:10.5713/ab.24.0892)
Supplement: Supplementary file 5 [file ab-24-0892-Supplementary-5.pdf]

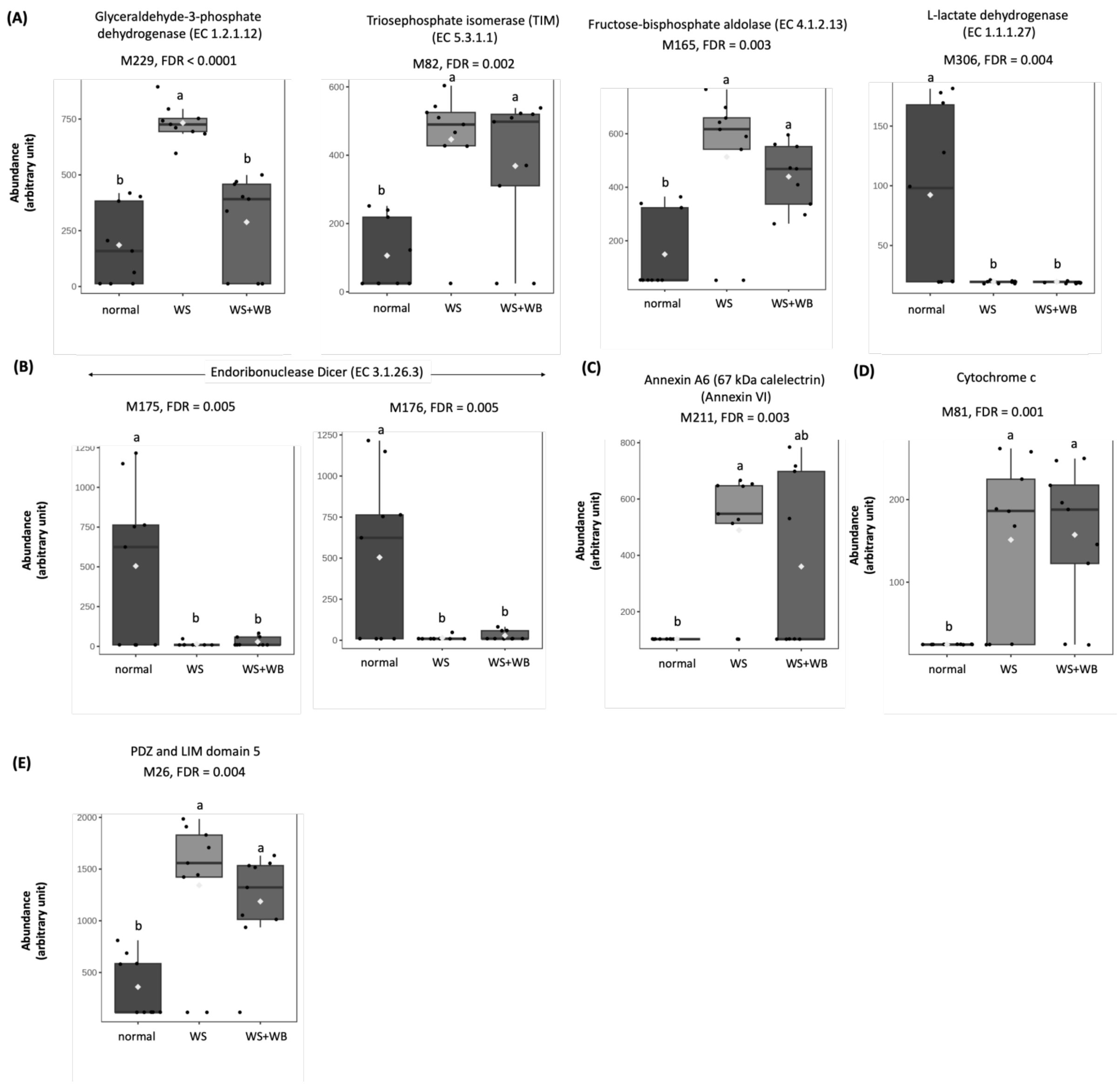

Supplement 5. Box plots illustrate average abundance (±standard deviation) of oxidative methionine (M) sites in proteins isolated from chicken meat exhibiting normal, wooden breast (WB), white striping (WS), and WS+WB. The proteins were related with (A) glycolytic enzymes, (B) post-transcriptional modification, (C) family of calcium-dependent membrane and phospholipid binding proteins, (D) oxidative respiration, and (E) regulatory proteins. FDR = false discovery rate, Different letters above bars indicate statistical significance (FDR < 0.05).
